# Resistance mechanisms and fitness of *Salmonella* Typhimurium and *Salmonella* Enteritidis mutants evolved under selection with ciprofloxacin *in vitro*

**DOI:** 10.1038/s41598-017-09151-y

**Published:** 2017-08-22

**Authors:** Chuan-Zhen Zhang, Si-Qi Ren, Man-Xia Chang, Pin-Xian Chen, Huan-Zhong Ding, Hong-Xia Jiang

**Affiliations:** 1 Guangdong Provincial Key Laboratory of Veterinary Pharmaceutics Development and Safety Evaluation, College of Veterinary Medicine, South China Agricultural University (SCAU), Guangzhou, 510642 China; 2 National Risk Assessment Laboratory for Antimicrobial Resistance of Animal Original Bacteria, College of Veterinary Medicine, South China Agricultural University (SCAU), Guangzhou, 510642 China

## Abstract

The aim of this study was to investigate the difference in resistance mechanisms and fitness of *Salmonella* Typhimurium (ST) and *Salmonella* Enteritidis (SE) mutants selected during the evolution of resistance under exposure to increasing ciprofloxacin concentrations *in vitro*. Mutations in quinolone target genes were screened by PCR. Phenotypic characterization included susceptibility testing by the broth dilution method, investigation of efflux activity and growth rate, and determination of the invasion of human intestinal epithelium cells *in vitro*. The two *Salmonella* serotypes exhibited differences in target gene mutations and efflux pump gene expression during the development of resistance. In the parental strains, ST had a competitive advantage over SE. During the development of resistance, initially, the SE strain was more competitive. However, once ciprofloxacin resistance was acquired, ST once again became the more competitive strain. In the absence of bile salts or at 0.1% bile, the growth rate of SE was initially greater than that of ST, but once ciprofloxacin resistance was acquired, ST had higher growth rates. ST strains showed decreased invasion of epithelial cells in 0.1% bile. These data indicate that ciprofloxacin-resistant ST strains are more competitive than ciprofloxacin-resistant SE strains.

## Introduction

*Salmonella* exhibit high morbidity and mortality, and are important pathogens worldwide. Host susceptibility and the serovar of the *Salmonella* strain determine how the infection is manifested^1^. According to the World Health Organization, *Salmonella* Typhimurium and *Salmonella* Enteritidis are the most frequently isolated *Salmonella* serotypes from countries involved in the Global Foodborne Infections Network^2^.

Fluoroquinolones (FQs) are the main drugs used for the treatment of salmonellosis. However, the emergence of FQ resistance has been reported in the past decade^3, 4^. Furthermore, recent reports have shown high isolation rates of FQ-resistant *Salmonella* strains from poultry samples^5^. FQ-resistance in Gram-negative bacteria is primarily due to the acquisition of mutations located in chromosomal genes encoding quinolone targets and, to a lesser extent, the overexpression of efflux pumps such as AcrAB-TolC or changes in outer membrane proteins (e.g., OmpF and OmpC)^6, 7^. Two enzymes are the main targets for the antibacterial activity of quinolones: DNA Gyrase (GyrA and GyrB) and topoisomerase IV (ParC and ParE). Amino acid substitutions at positions 83 and 87 of GyrA are the most frequently reported mutations in FQ-resistant isolates^8, 9^.

Chromosomally mediated FQ resistance mutations have been reported to be associated with decreased fitness^10^. There is not always a link between resistance and fitness costs and, additionally, bacteria may reverse fitness costs by acquiring compensatory mutations. Moreover, fitness is an important parameter for evaluating the epidemiological features of a given microorganism; resistance mechanisms can have varied effects on the organism^11, 12^, which affect its ability to survive in the presence of other bacterial populations once antibiotic pressure is removed^13^.

Upon colonization in the intestine, virulent *Salmonella* strains localize to the apical epithelium and induce invasion-associated virulence mechanisms^14^. During the pathogenic process, *Salmonella* encounters bile, an antimicrobial, in the gastrointestinal tract. Once exposed to subinhibitory concentrations of bile, the expression of numerous genes related to the invasion of intestinal cells and resistance to bile change^15^. In addition, the *acrAB* and *tolC* genes, which encode the major multidrug AcrAB-TolC efflux system, also required for the bile resistance of *Salmonella*, are transcriptionally activated by bile^16^.

We found that a significant percentage of FQ-resistant isolates from *Salmonella* Typhimurium simultaneously presented resistance to cephalosporins and other commonly used antibiotics in food producing animals between 2010 and 2015^17, 18^. When developing resistance, certain *Salmonella* serovars showed fitness benefits, while others showed a fitness cost; thus, there was a persistent distribution of a serovar. *Salmonella* Enteritidis and Typhimurium are the most common serotypes in Europe from patients (European Centre for Disease Prevention and Control, 2013), although there are rising reports of *Salmonella* Indiana being the most prevalent serotype in food producing animals in China besides *Salmonella* Typhimurium, rather than *Salmonella* Enteritidis^5, 18^. Thus, we hypothesized that FQ-resistant *Salmonella* Typhimurium and Indiana have fitness benefits that contribute to the persistence of these serovars. The main objective of this study was to investigate and compare differential fitness between a collection of *Salmonella* Typhimurium and *Salmonella* Enteritidis mutants selected *in vitro*. Fitness assessments included FQ resistance mechanisms, bacterial growth, and growth competition. Additionally, we assessed bacterial growth and invasion abilities on exposure to different concentrations of bile salts.

## Results

### Characteristics of quinolone resistance-determining region (QRDR) mutations in the two serotypes during ***in vitro*** selection for resistance to quinolones

The two *Salmonella* serotypes exhibited different target gene mutations during the development of resistance. Considering ST, mutants ST-M1 and ST-M2 had a reduced susceptibility to ciprofloxacin. The mutation G81D in GyrA was detected in ST-M2. When the ciprofloxacin concentration increased to 2 mg/L, mutant ST-M3 acquired high-level resistance to ciprofloxacin; an additional mutation in ParC (G78D) was detected, but this was subsequently restored with further increases in ciprofloxacin concentration, leading to a fourfold decrease in the ciprofloxacin MIC in the subsequent mutants (ST-M4, ST-M5, ST-M6). To determine the role of mutation ParC G78D in ciprofloxacin resistance, we examined the effect of complementation with the parental strain *parC* gene by determining antibiotic susceptibilities (data not shown). These experiments showed that the G78D mutation in ParC was responsible for increased resistance to fluoroquinolones, rather than nalidixic acid, which is in accordance with the results of a previous study^19^.

SE mutant SE-M1 had reduced susceptibility to ciprofloxacin (MIC = 0.125 mg/L) relative to the parental strain that accompanied a S464F mutation in GyrB. No additional mutations were detected in the subsequent mutants (SE-M2, SE-M3, SE-M4, and SE-M5) that became ciprofloxacin-resistant. A mutant with high-level resistance, SE-M6, displayed additional mutations in GyrA (D87A) and ParE (V461G) (Table 1).

**Table 1:** Antimicrobial susceptibility and mutations in target genes of parental strains and their respective mutants.

strain	CIP pressure (mg/L)	MIC(mg/L)						Amino acid substitutions			
		CIP	ENR	LEV	OFL	NOR	NAL	GyrA	GyrB	ParC	ParE
**ST**		**0.03**	**0.03**	**0.03**	**0.06**	**0.06**	**8**	—	—	—	—
ST-M1	0.125	0.125	0.06	0.125	0.125	0.125	8	—	—	—	—
ST-M2	0.25	0.25	1	1	1	1	32	G81D	—	—	—
ST-M3	2	16	8	32	128	32	32	G81D	—	G78D	—
ST-M4	4	4	8	8	8	16	64	G81D	—	—	—
ST-M5	16	4	8	4	8	8	64	G81D	—	—	—
ST-M6	32	4	8	8	16	32	128	G81D	—	—	—
**SE**		**0.03**	**0.03**	**0.06**	**0.06**	**0.06**	**8**	—	—	—	—
SE-M1	0.125	0.125	0.125	0.25	0.5	0.5	32	—	S464F	—	—
SE-M2	0.25	0.25	0.25	0.25	1	1	64	—	S464F	—	—
SE-M3	2	2	2	2	2	8	256	—	S464F	—	—
SE-M4	4	4	4	4	8	16	512	—	S464F	—	—
SE-M5	16	4	4	4	8	16	128	—	S464F	—	—
SE-M6	32	32	32	32	32	128	>512	D87A	S464F	—	V461G

ST: *Salmonella* Typhimurium; SE: *Salmonella* Enteritidis.

M1, M2, M3, M4: serial selected mutants; **-**: no mutation.

CIP: ciprofloxacin; ENR: enofloxacin; LEV: levofloxacin; OFL: ofloxacin; NOR: norfloxacin; NAL: nalidixic acid; TET: tetracycline.

### Growth curves of mutants derived from SE or ST

**Figure 1:**
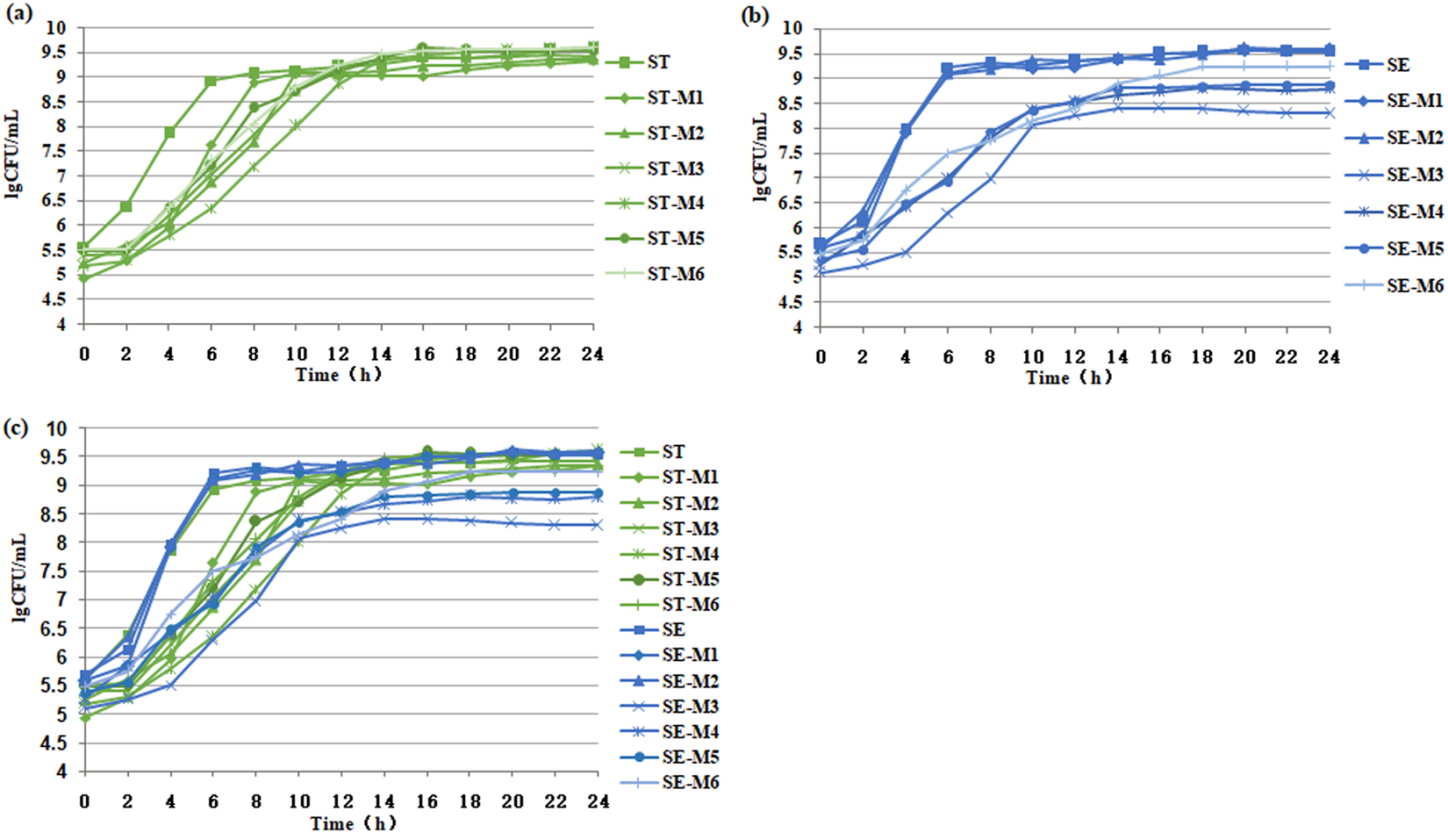
Bacterial growth curves of: (**a**) ST and its mutants; (**b**) SE and its mutants, and; (**c**) ST, SE and their corresponding mutants in antibiotic-free LB broth. Results are the mean of three independent experiments.

ST mutants had a much longer lag phase than the parental strain; however, once mutants reached the stationary phase (~12 h), there were negligible differences between the mutants and the parental strain (Fig. 1a). SE mutants (SE-M1, SE-M2), which presented reduced susceptibility to ciprofloxacin, exhibited the same growth as the parental strain; however, the mutants (SE-M4, SE-M5, and SE-M6) that acquired resistance had longer lag phases than the parental strain and did not reach the same cell density in the stationary phase (Fig. 1b). SE-M6, which contained additional two mutations (in GyrA and ParE), grew better than the other mutants. The growth of mutant SE-M3, which exhibited intermediate resistance to ciprofloxacin (MIC = 2 mg/L), was greatly impaired, even from the initial phase, with much lower CFU counts than the other mutants. There were no significant differences between the growth rate of the two different serotype parental strains (ST and SE); however, there were differences in the growth rates of the mutants. The growth rate of SE mutant obtained under initial antibiotic pressure was significantly greater than that of ST mutant; however, the growth rates of ST mutants obtained under increased and sustained antibiotic pressure was significantly greater than that of SE mutants (Fig. 1c).

### Expression of efflux-related genes

Mutants exhibiting reduced antibiotic susceptibility (ST-M1 and SE-M1), resistance (ST-M4 and SE-M4, ST-M5 and SE-M5, and ST-M6), and high-level resistance (SE-M6) were selected for analysis of *acrA*, *acrB*, *tolC*, *ramA*, *marA*, *soxS*, and *ompF* gene expression (Table 2). ST mutants had higher expression levels of efflux pump genes compared with the parental strain. However, the efflux pump genes were not activated in SE mutants compared with the parental strain. The expression levels of the positive transcriptional regulator *ramA* in ST mutants were much higher than in the SE mutants; meanwhile, there was decreased expression of *marA* and *soxS* in ST mutants compared with the parental ST. The expression of *ompF* was decreased in ST-M4 and ST-M5 compared with the parental ST.

**Table 2:** Mean (SD) values of RT-PCR analysis obtained in six independent experiments.

Strain	Gene expression values						
	*acrA*	*acrB*	*tolC*	*ramA*	*marA*	*soxS*	*ompF*
SE	1	1	1	1	1	1	1
SE-M1	1.3(0.7)	1.5(0.9)	1.9(1.7)	1.4(0.5)	1.6(0.7)	2.1(1.4)	1.7(1.6)
SE-M4	1.1(0.6)	1.2(0.5)	1.4(1.1)	1.5(1.8)	1.5(1.3)	−1.1(1.2)	−1.4(0.3)
SE-M5	1.1(0.6)	1.3(0.6)	1.8(1.2)	−1.1(0.4)	2.1(1.4)	1.3(0.6)	1.6(1.4)
SE-M6	1.1(0.5)	1.2(0.6)	1.4(0.9)	1.2(0.9)	2(2.1)	1.6(1.8)	1.6(1.3)
ST	1	1	1	1	1	1	1
ST-M1	−1.7(0.3)	−1.4(0.1)	−1.3(0.2)	−1.3(0.6)	1.2(0.5)	−1.1(0.2)	3.1(2.6)
ST-M4	1.3(0.5)	2.8(1.3)	1(0.5)	14.2(3.6)	−1.7(0.2)	−3.3(0.07)	−5(0.2)
ST-M5	2.4(1.2)	3.3(1.5)	1.8(0.7)	8.3(3.2)	−2(0.1)	−2.5(0.03)	−1.3(0.8)
ST-M6	1.8(0.7)	3.3(1.3)	2.6(1.1)	29.3(15.2)	1.5(0.8)	−1.4(0.1)	1(0.5)

### Growth competition assays by pyrosequencing

We assessed the competitive advantages and disadvantages of the parental strains (ST and SE) as well as three mutants (M2, M3, and M6), which contained various QRDR mutations. The ability to differentiate strains by bacterial culture on selective media is limited; thus, we developed four pyrosequencing assays to distinguish between the two serotype strains at the same ciprofloxacin concentrations based on single nucleotide variations in *gyrA*.

**Figure 2:**
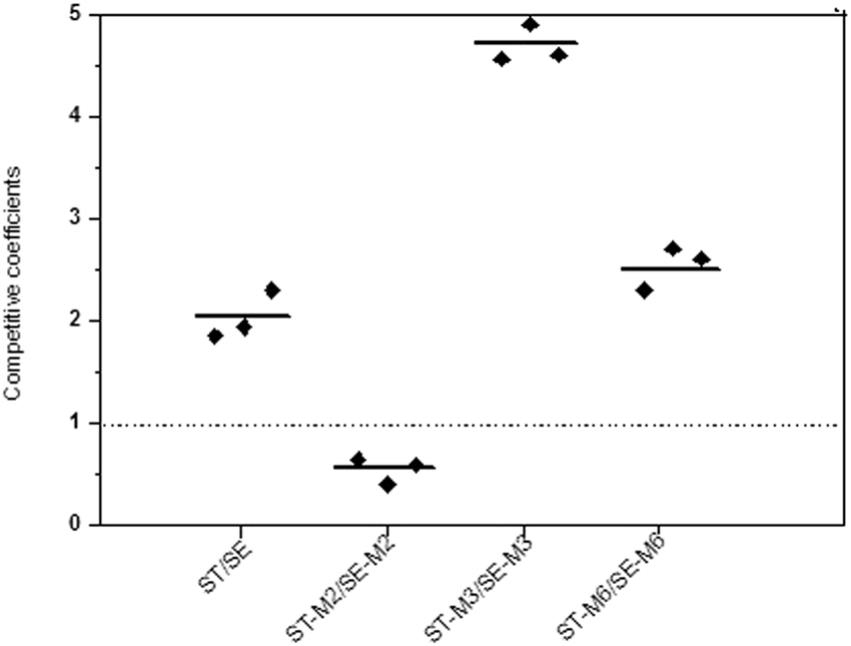
*In vitro* bacterial competition assays. Competition coefficient values obtained from each independent experiment are plotted; a competition coefficient of 1 is noted by a broken horizontal line, and means are noted by a short continuous horizontal line.

For the two parental strains, the competition coefficient between ST and SE was 1.9; this indicates that ST had a competitive advantage. During the development of resistance, initially, the SE mutant was more competitive (the competition coefficient between ST-M2 and SE-M2 was 0.6). However, once ciprofloxacin resistance was acquired, the ST strain once again became the more competitive strain (the competition coefficient between ST-M3 and SE-M3 was 4.6). When the resistance increased to high levels, the ST strain remained the more competitive strain (the competition coefficient between ST-M6 and SE-M6 was 2.7) (Fig. 2).

This result indicated that ciprofloxacin-resistant ST strains have competitive growth advantages over ciprofloxacin-resistant SE strains.

### Bile tolerance

**Figure 3:**
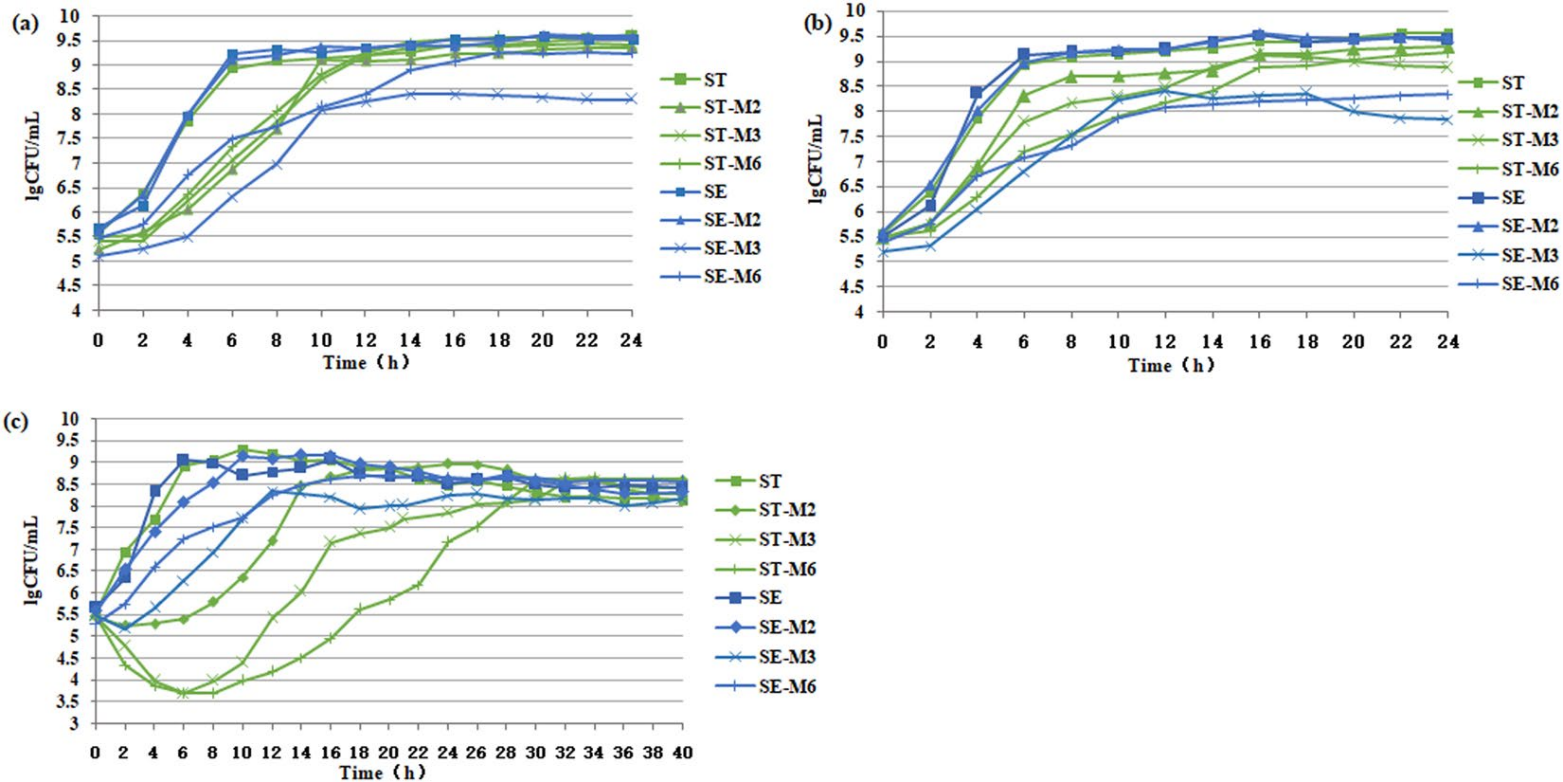
Bile tolerance. The strains were grown in the presence of: (**a**) no bile, (**b**) 0.1% bile, and (**c**) 1% bile.

We measured bile tolerance by examining bacterial growth at various bile concentrations (Fig. 3). In the absence of bile salts, the two parental strains exhibited similar growth characteristics. During the development of resistance, SE initially exhibited higher growth rate than ST; however, at higher resistance levels, the growth rate of ST was greater than that of SE (Fig. 3a). In the presence of bile salts, the growth rate of both ST and SE was reduced. During the development of resistance, when the strains showed reduced susceptibility to ciprofloxacin, SE had higher growth rate than ST; however, the growth rate of ST became greater than that of SE following the acquisition of resistance to ciprofloxacin at the low bile salt concentration (0.1%) (Fig. 3b). However, SE had higher growth rate than ST throughout the growth at the high bile salt concentration (1%) (Fig. 3c).

### Invasion ability of *Salmonella* strains

**Figure 4:**
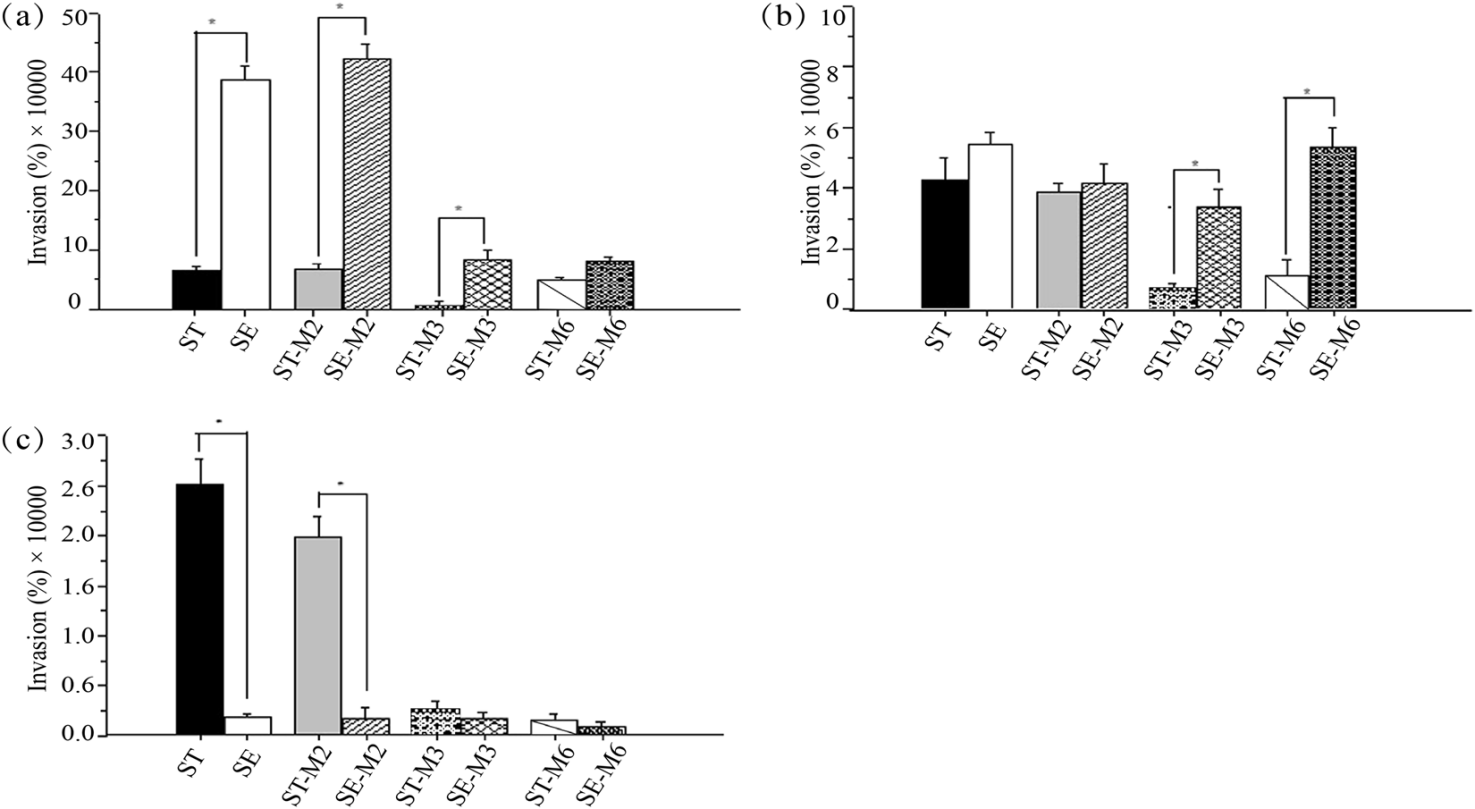
Ability of *Salmonella* treated at different concentrations of bile salts to invade INT-407 epithelial cells: (**a**) no bile, (**b**) 0.1% bile, (**c**) 1% bile. Results are the mean of three independent experiments ± standard error. Differences between groups were assessed using the one-way ANOVA test. *P < 0.05.

In some *Salmonella* serovars, acquisition of quinolone resistance has been related to decreased ability to invade eukaryotic cells^20, 21^. Furthermore, bile has been shown to impact the invasion process^22^. To determine if there were any differences in the invasion ability of the two serotypes and their respective mutants, we examined the ability of above strains exposed to different bile concentrations (0%, 0.1%, 1%) to invade INT-407 cells *in vitro*. Results are expressed as the percentage of the inoculum that effectively invaded the INT-407 cells. In absence of bile salts, the ST parental strain and mutants adhered to INT-407 cells significantly less than the SE parental strain and mutants, with the exception of the high-level ciprofloxacin resistant mutants ST-M6 and SE-M6, which had similar adherence (Fig. 4a). At the low bile salt (0.1%) concentration (Fig. 4b), the two parental strains and their respective reduced susceptibility mutants (ST-M2 and SE-M2) had similar adherence; meanwhile, the resistant and high-level resistant ST mutants had significantly lower adherence than the SE mutants. At the highest bile salt concentration (1%) (Fig. 4c), the parental strain ST and its reduced susceptibility mutant had higher adherence than the parental strain SE and its reduced susceptibility mutant; meanwhile, the resistant and high-level resistant mutants of both ST and SE had decreased adherence.

## Discussion

In this study we observed differences in the evolution of resistance between two *Salmonella* serotypes under stepwise selection with increasing ciprofloxacin concentrations. For ST, the first GyrA mutation (G81D) was associated with a reduced susceptibility to ciprofloxacin. Mutations in GyrA residue 81 [glycine (Gly) to either cysteine (Cys) or histidine (His)] in *Salmonella* spp. have been associated with resistance to fluoroquinolones; however, alteration from Gly to aspartic acid (Asp) has only been reported in *E. coli*, where it is responsible for the development of quinolone resistance^23^. Subsequently, another mutation (G78D in ParC) occurred that led to high-level ciprofloxacin resistance (MIC = 16 mg/L). However, this mutation was lost, and subsequently high-level resistance was not retained even under higher ciprofloxacin pressure. The loss of the mutation presumably occurred when the mutant was passaged in ciprofloxacin-free LB broth. For SE, occurrence of the first mutation in GyrB (S464F) was accompanied by reduced susceptibility to ciprofloxacin. No additional mutations occurred; however, the strain became ciprofloxacin-resistant under increasing drug pressure. Under persistent high drug pressure, the strain evolved high-level ciprofloxacin resistance that was accompanied by additional mutations in GyrA and ParE. Substitutions in GyrB and ParE have been reported to be much less prevalent and to only make a minor contribution to resistance^24^.

Different mutation patterns in quinolone target genes occurring in different *Salmonella* serotypes during the evolution of resistance were also found in our previous study^25^. In that study, first and subsequent mutations were detected in GyrA (D87G) and GyrB (E466D), respectively, which played a major role during the whole resistance development of SE; however, in ST, the first mutation was detected in GyrA (D87N) and an additional mutation in GyrB was only found in mutants with high-level resistance^25^. Both of these independent studies (the present and previous study^25^) demonstrated that mutations in GyrA played an important role in the evolution of resistance in ST; however, mutations in GyrB contributed more during evolved resistance of SE. The minor differences in the mutations that occurred in these two studies probably arose from: (i) the culture medium (LB medium in the present study vs. MH medium in the previous study); (ii) the selected ciprofloxacin pressure (starting from ciprofloxacin concentration equal to the MIC in the present study vs. starting from half of the ciprofloxacin MIC in the previous study); and (iii) that mutants were incubated in LB broth without ciprofloxacin in the present study, whereas in the previous study they were incubated in MH broth containing the MIC of ciprofloxacin before the next round of selection. Based on these outcomes, we speculate that in the development of ciprofloxacin resistance, chromosomal mutations of the target gene *gyrA* are the main contributor for ST; meanwhile, mutation in *gyrB* along with mutation in *gyrA* are presumably the main contributors to resistance in SE.

The expression of AcrAB-TolC is a compensatory mechanism in the evolution of resistance. Several studies have reported that, in *Salmonella*, the first and primary step towards a resistance phenotype is the acquisition of mutations that increase efflux, mainly due to the overexpression of AcrAB. Mutations in the QRDRs represent the second step, along with other mutations that contribute to increased efflux activity^20, 26^. In the present study, overexpression of the major efflux pump AcrAB-TolC was detected in ST mutants. There was a reduction in *ompF* expression in resistant mutants (ST-M4 and ST-M5), which is known to confer low-level quinolone resistance^27^. However, there was no overexpression of AcrAB-TolC in the SE mutants. These findings are in accordance with previous study showing that: (i) in SE, target gene mutations in GyrA and GyrB are important in the development of ciprofloxacin resistance; and (ii) in ST, the AcrAB-TolC efflux pump had a crucial impact on the whole resistance-development process^25^.

The difference in resistance mechanisms between the two *Salmonella* serotypes corresponded to different growth rates; mutations in GyrA and ParC in ST in the initial proliferation phase impaired growth, but were ultimately beneficial in the plateau phase where the mutant grew better than the parental strain. Mutations in target genes are beneficial to bacterial growth during resistance evolution. Baker *et al*. showed that double mutants in GyrA (position 83 or 87) and ParC (position 80) in *Salmonella* Typhi exhibited higher fitness than expected as a result of synergistic epistasis, conferring a measurable fitness advantage over strains without these mutations^28^. However, in the present study, the fitness cost produced by GyrB mutation was only partially compensated by additional mutations in GyrA and ParE in SE. Similarly, double mutants in GyrA (position 81) and ParC (position 78) did not exhibit higher fitness than the single ST GyrA mutant. We hypothesize that strong synergistic epistasis is greatly dependent on hot gene mutations, e.g. GyrA and ParC rather than GyrB, as well as hot mutation sites, e.g. GyrA*-*83/87 rather than GyrA-81, and ParC*-*80 rather than ParC*-*78. Furthermore, the mutant strains used in this study were not isogenic; it should be taken into consideration that other genetic changes in the mutants might produce the observed phenotypic differences.

From the results of mutations detected in target genes, growth competition, and the expression of *acrAB*, we can speculate about the evolutionary process of acquisition of resistance by ST and SE while exposed to ciprofloxacin and the fitness of these organisms. In the early stage of resistance development, a first mutation occurred in a target gene in both of the strains which led to reduced susceptibility to ciprofloxacin but a difference in fitness: the mutation in ST caused a fitness cost, while the mutation in SE enhanced the fitness. Thus, the SE mutant had a competitive advantage. Once it acquired resistance, particularly high-level resistance, the additional mutation in ParC (G78D) gave ST a competitive advantage over SE. Furthermore, multiple mutations in target genes produced a burden, impairing the growth of SE. This may explain the prevalence of ST among food produced on animal farms where fluoroquinolones are extensively used. However, limitations of our experiments are that they were performed in laboratory (non-restricted) conditions. We must perform experiments in natural environments (low temperature, low water activity, inhibitory substances) or in warm-blooded hosts (nutrient restriction, possible pH effects, etc.), because mutations compensating for fitness cost *in vitro* are different to those *in vivo* ^29^.

*Salmonella* causes gastrointestinal infections and normally enters the intestinal tract after being consumed in contaminated foods. As bile is released into the digestive tract to aid in digestion of lipids, *Salmonella* is exposed to these stressful compounds. The AcrAB-TolC efflux pump is required for growth of *Salmonella* in the presence of bile; however, overexpression of efflux pumps is usually associated with decreased fitness^16^. This may account for the fact that the ciprofloxacin-susceptible strains were more resistant to bile salts than the ciprofloxacin-resistant strains, as well as the fact that the resistant ST strains had worse growth rates due to overexpression of efflux activity than the resistant SE strains when exposed to high bile salt concentration (1%).

Studies conducted with SE and ST showed an association between resistance and reduced virulence in quinolone-resistant mutants^30, 31^. Consistent with these findings, our results show that the resistant mutants have a reduced ability to invade epithelial cells. Bile mediates the activation of *acrAB* and induces high levels of AcrAB-TolC production, which can lead to impaired virulence^21^. This may account for the reduced invasion ability seen following exposure to bile salts. In addition, bile also represses virulence genes in *Salmonella*. *Salmonella* grown in the presence of bile demonstrated a drastic repression of invasion genes; as suspected, this corresponded to a decrease in the ability to invade epithelial cells. It has been speculated that the ability of enteric bacteria to survive in the presence of large quantities of bile salts is directly related to their ability to establish invasive infections in the intestine^32^. Nonetheless, our results do not support this hypothesis, since resistant ST strains had higher growth but decreased invasion of epithelial cells at 0.1% bile.

In conclusion, the two most commonly reported *Salmonella* serovars, SE and ST, have different resistance mechanisms during the evolution of resistance under drug pressure. This led to differences in growth rates. Ciprofloxacin-resistant ST strains were more competitive than ciprofloxacin-resistant SE strains; this could explain why ST is the predominant multidrug-resistant serovar.

## Materials and Methods

### Bacterial strains and selection of resistant mutants

ST and SE were isolated from the rectal swabs of two individual chickens. The strains were susceptible to quinolones (i.e., no mutations within QRDRs of the four target genes as determined by PCR) and commonly used drugs. These were used as the two parental strains. Strains were grown at 37 °C on LB agar plates. Ciprofloxacin (Fluka) was only present during selection, which started at 0.03 mg/L (equal to the MIC for ST and SE) and increased twofold to a maximum concentration of 32 mg/L. The mutants presented here were selected at 0.125, 0.25, 2, 4, 16, and 32 mg/L ciprofloxacin, and were named M1, M2, M3, M4, M5, and M6, respectively. The mutants from each procedure were passaged five times in LB broth without ciprofloxacin. All ST and SE mutants were grouped according to MICs as follows: reduced susceptibility (MIC = 0.5–2 mg/L), resistance (MIC = 4–8 mg/L) and high-level resistance (MIC ≥ 16 mg/L), according to CLSI guidelines. No live vertebrates were included in this study and all strains were manipulated in a biohazard containment 2 facility. All waste cultures were inactivated to prevent infection or release into the surrounding environment.

### Antimicrobial susceptibility

MICs were determined in triplicate for each bacterial strain using broth microdilutions according to the CLSI reference methods. The following antimicrobials were tested: ciprofloxacin (CIP), nalidixic acid (NAL), enrofloxacin (ENR), levofloxacin (LEV), ofloxacin (OFL), and norfloxacin (NOR). *Escherichia coli* ATCC 25922 was used as the control strain.

### Detection of mutations within QRDRs

Mutations acquired in the QRDR genes GyrA, GyrB, ParC, and ParE were detected by PCR^33^. All PCR products were directly sequenced, and the results were compared with the genome of *Salmonella* Typhimurium LT2 (Ref Seq: NC_003197.1).

### Bacterial growth

Strains were incubated overnight in Luria-Bertani (LB) broth at 37 °C with shaking, and then diluted to OD_600_ = 0.1. This was diluted 1:500 in fresh LB broth and incubated with shaking (200 rpm). At 2-h intervals, samples were serially diluted and plated onto LB agar plates to estimate growth. The plates were incubated at 37 °C for 24 h before counting colonies. Three independent assays were performed for each strain, and the standard deviation was within 10%.

### RNA extraction and real-time PCR

RNA extraction from exponential cultures of the two parental strains (ST and SE) and three mutants (M1, M4, and M5) was performed according to Fàbrega *et al*.^30^. The expression of *acrA*, *acrB*, *tolC*, *ramA*, *marA*, and *soxS* genes was analyzed by RT-PCR^34^. The 16S rRNA gene was used as an internal control for normalization, and the parental strains were used as references for their derived mutants. The 2^−ΔΔCT^ method was used for relative gene expression calculations. Five independent assays were performed, and each RNA sample was tested in triplicate. The primers used are listed in Table 3.

**Table 3:** Primers used for real-time PCR.

Gene	Primers (5′ to 3′)
**Efflux and permeability components**	
*acrA*	*acrA*-F CCCGGATCACACCTTATTGC
	*acrA*-R CTGGCTTGCGACGATTTG
*acrB*	*acrB*-F TTGCAGGGCGCGGTCAGAATAC
	*acrB*-R TGCGGTGCCCAGCTCAACGAT
*tolC*	*tolC*-F GTGACCGCCCGCAACAAC
	*tolC*-R ATTCAGCGTCGGCAGGTGAC
*ompF*	*ompF*-F GGGCGCGACTTACTACTTCAAC
	*ompF*-R TCGTTTTCGTCCAGCAGGTT
**Regulatory genes**	
*soxS*	*soxS*-F AAATCGGGCTACTCCAAGTG
	*soxS*-R CTACAGGCGGTGACGGTAAT
*marA*	*marA*-F ATCCGCAGCCGTAAAATGAC
	*marA*-R TGGTTCAGCGGCAGCATATA
*ramA*	*ramA*-F CACGATTGTCGAGTGGATTG
	*ramA*-R AAAATGCGCGTAAAGGTTTG
**Reference gene**	
*16S rRNA*	16S-F GCGGCAGGCCTAACACAT
	16S-R GCAAGAGGCCCGAACGTC

### Growth competition assays by pyrosequencing

We tested growth competition between the two parental strains (i.e., ST vs. SE), as well as three pairs of mutants (i.e., ST-M2 vs. SE-M2, ST-M3 vs. SE-M3, and ST-M6 vs. SE-M6) in antibiotic-free LB broth. Each test was run with five replicates. Growth competition was determined by pyrosequencing the single nucleotide variations in GyrA of the strains according to the method of Baker *et al*.^28^. Bacterial cells from the mixed culture (1:1 ratio) were thawed, and DNA was extracted using heat treatment and phenol-chloroform purification. Briefly, a 100 µL aliquot of the mixed culture was agitated and incubated at 100 °C for 10 min before being returned to ambient temperature. The solution was centrifuged, an equal volume of phenol-chloroform was added, the mixture was then vortexed and centrifuged at 10,000 × *g* for 20 min in a benchtop microfuge (Eppendorf, USA). The supernatant was removed, placed in a sterile microfuge tube, and immediately prepared for pyrosequencing^28^. The DNA from the competitive growth assays was PCR amplified in triplicate using biotinylated primer pairs targeting the region containing the single nucleotide polymorphism to distinguish the two organisms in the assay by mutations in GyrA. All PCR amplifications were visualized on 1% agarose gels prior to pyrosequencing. The purified PCR products were pyrosequenced at the BGI Company.

Competition coefficients were defined as the ratio of the ST strain to the SE strain, and were calculated by measuring the percentage yield of the single nucleotide in *gyrA*. A competition coefficient >1 means that the ST strain was the more abundant strain; meanwhile, a competition coefficient <1 means that the ST strain was the less abundant strain.

### Bile tolerance

Independent overnight cultures were made for the parental strains and selected mutants. The bacteria were diluted 1:500 in LB medium containing 0%, 0.1%, or 1% bile (Fluka Bile Salts, Sigma)^35^. The growth of the bacteria was measured by counting viable colonies every 2 h for 24 h (40 h for 1% bile) at 37 °C. The experiment was repeated three times, and the results were averaged for each strain.

### Cell invasion assays

A human intestinal epithelium cell line (INT-407) was cultured at 37 °C under 5% CO_2_ in Dulbecco’s Modified Eagle’s Medium (DMEM; HyClone Laboratories Inc., Logan, UT, USA) containing 10% fetal bovine serum (FBS) and 100 mg/L gentamicin. The cultured cells were seeded (10^6^ cells/mL) into 24-well tissue culture plates (BD Falcon, Franklin Lakes, NJ, USA) to reach >90% confluence at 37 °C. The post-confluent cultures were rinsed twice with PBS and preincubated in prewarmed antibiotic-free DMEM for 2 h. After stabilization, the INT-407 cell monolayers were used to evaluate the invasive ability of ST and SE cells exposed to different bile conditions. ST and SE cells exposed to 0%, 0.1%, or 1% bile were suspended in antibiotic-free DMEM (approximately 8.0 × 10^5^ CFU/mL) and infected into the INT-407 cell monolayers at 37 °C for 2 h. After infection, the INT-407 cell monolayers were incubated at 37 °C for 2 h in DMEM containing 100 mg/L gentamicin to inactivate extracellular ST and SE cells, rinsed with PBS to eliminate the gentamicin residue, and lysed with 1% Triton X-100 for 15 min at 37 °C. The lysates were serially diluted (1:10) with PBS. The proper dilutions were plated on LB-agar and incubated at 37 °C for 24–48 h, and intracellular ST and SE cells were counted^36^. Each invasion assay was performed at least three times (four wells per assay).

### Statistics

Data were analyzed using IBM SPSS Statistics 20. The data from cell invasion assays were normally distributed; thus, multiple comparisons were performed using a one-way ANOVA test. P-values < 0.05 were considered to be significant.
